# Effects of node number of vine cuttings and planting methods on endogenous hormone contents at nodes and storage root formation in sweet potato

**DOI:** 10.1371/journal.pone.0351031

**Published:** 2026-07-08

**Authors:** Yiming Song, Zhiqiang He, Guangyan Sun, Shuwen Deng, Zhenpeng Deng, Changwen Lyv, Daobin Tang, Jichun Wang

**Affiliations:** 1 Yibin Academy of Southwest University, Yibin, Sichuan, China; 2 College of Agronomy and Biotechnology, Southwest University / Chongqing Key Laboratory of Biology and Genetic Breeding for Tuber and Root Crops, Chongqing, China; University of the West Indies at Cave Hill, BARBADOS

## Abstract

As a globally significant dual-purpose crop, sweet potato yield is regulated by the node number of vine cuttings and the planting method. Elucidating the effects of stem segment number and soil-entry method on endogenous hormones within sweet potato nodes and storage root formation holds significant scientific value, providing theoretical basis and technical support for optimizing planting techniques and enhancing yields. A two-factor split-plot design was implemented from 2022 to 2023 using the Yuhongxinshu 98 cultivar. Main plots tested vine cuttings with 6 (A1), 8 (A2), or 10 nodes (A3). Subplots compared slanted planting with exposed tip (B1), flat planting with exposed tip (B2), and flat planting without exposed tip (B3). The study investigated the effects of these treatments on growth hormone content at nodes, root morphology, and yield. Results indicated that increasing vine cutting node number to 8 and 10 significantly enhanced early adventitious root development, including root length, root diameter, and root tip number, and increased aboveground branching. Compared with 6-node cuttings, 8- and 10-node cuttings increased storage root yield by 23.43% and 30.03%, respectively. Compared with slanting planting with exposed tip, flat planting with exposed tip and flat planting without exposed tip increased storage root yield by 20.11% and 18.10%, respectively. At 70 DAT, corresponding to the early storage root enlargement stage, ABA and IAA contents in buried nodes increased with vine cutting node number. IAA content showed a node-dependent pattern and peaked at the fourth buried node, whereas ABA content was highest at the second buried node. Correlation analysis showed that ABA and IAA contents were associated with storage root yield, storage root number, and root morphological traits. Nodal analysis indicated that storage root formation was mainly concentrated in the upper buried nodes, especially the second buried node, while flat planting increased the contribution of multiple buried nodes to storage root formation. Overall, 8-node vine cuttings combined with flat planting, especially A2B2 and A2B3, showed better performance in coordinating early root establishment, nodal storage root formation, and yield improvement. These findings provide a practical basis for optimizing sweet potato planting techniques through improved vine cutting node number and planting method.

## 1. Introduction

Sweet potato (*Ipomoea batatas* L.) is a globally significant food, feed, and industrial raw material crop. Its yield depends on the storage roots formed at various nodes along the underground vines [1–3]. Planting marks the initial stage of sweet potato production, where the node number of vine cuttings used and planting method directly determine the quantity, spatial distribution, and microenvironment of the initial soil-entry nodes. This constitutes a crucial agronomic practice influencing the storage capacity and activity of each node [4–7].

Studies have shown that the differentiation of adventitious roots in sweet potatoes during the early stage significantly influences storage root formation capacity at different nodes along the vines [8–10].The growth of adventitious roots during the initial 20–40 days influences tuber differentiation direction, directly determining their potential as effective storage organs [9,11].Simultaneously, influenced by proximity to nutrient supply, underground nodes closer to the aboveground photosynthetic source exhibit more branching and gain a competitive advantage in assimilate acquisition [12–13]. Research indicates that increasing the node number of vine cuttings from 5 to 7–9 [7–8]or using 6-node seedlings for flat planting [2,3,6]significantly boosts the number of storage roots per unit area and total yield.

Numerous studies indicate that hormone concentrations in culture media influence rooting and cambium activity in tissue-cultured seedlings, while leaf hormone dynamics affect tuber formation and growth [4,9,10]. Endogenous IAA in underground vine nodes promotes tuber initiation and enlargement [14–15], while endogenous ABA in underground vine nodes participates in tuber induction, enhances enzyme activity related to starch synthesis, and expands storage capacity [16–19]. The concentration and variation of endogenous hormones like IAA and ABA across different underground nodes constitute the intrinsic physiological basis for their storage capacity differentiation [10,20].

In summary, the node number of vine cuttings directly influences tuber formation and is also affected by hormone levels in the plant’s leaves and roots. However, in production, varying numbers of planted stem segments and planting methods significantly impact the node number of vine cuttings buried underground. The effects of these buried nodes on tuber formation and their underlying hormonal mechanisms remain largely unreported and warrant further investigation.

Therefore, this study was designed to investigate the dynamic changes in hormone content at different nodes of underground vines, root system morphology, and aboveground vine growth in sweet potato under different node numbers of vine cuttings and planting methods. Through elucidating the nodal characteristics and hormonal regulation mechanisms of storage root formation, this research seeks to provide guidance for optimizing the node number of vine cuttings and planting methods for high-yield sweet potato production, offering valuable theoretical and practical insights.

## 2. Materials and methods

### 2.1. Experimental site and cultivar

**Fig 1:**
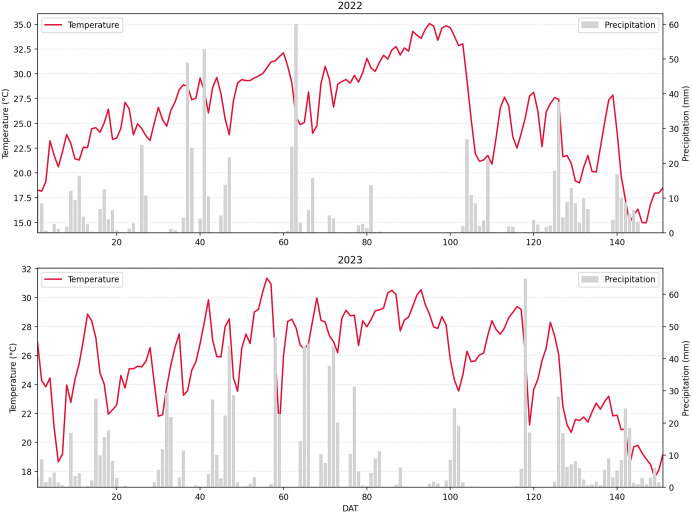
Temperature and precipitation during transplanting period from 2022 to 2023. (A) This is the legend for temperature and precipitation during transplanting period from 2022 to 2023.

The experimental site is located at the Southwest University Experimental Farm in Weituo Town, Hechuan District, Chongqing (30°04'E, 106°15'N). The test soil is sandy loam, with basic nutrients in the topsoil shown in Table 1. The test material was Yuhongxinshu 98, a fresh-eating sweet potato cultivar widely promoted in the Southwest China region. It exhibits strong storage root formation ability, with β-carotene content around 6 mg·100g^-1^ FW and starch content around 18%. The average tuber yield per plant is 5–6 tubers. The material was provided by the Chongqing Key Laboratory of Tuber Biology and Genetic Breeding, Southwest University. Compared with other typical sweet potato cultivars, Yuhongxinshu 98 adopted in this study presents highly competitive yield performance in Southwest China, especially among fresh-eating sweet potato varieties. Under the conventional planting density of 45000–60000 plants per hectare in this region, its average fresh tuber yield exceeds 2600 kg per mu, and the average single plant fresh weight ranges from 0.65 kg to 0.85 kg. The planting materials used in the experiment were healthy apical cuttings free of pests and diseases. At the time of cutting, the seedling age was approximately 35 days, with an average stem diameter of 5.0 mm. During the sweet potato growing seasons (from May to October) in 2022 and 2023, key meteorological data, including monthly average temperature and total precipitation, were recorded by the local meteorological station (Fig 1). The average temperatures were approximately 26.17 °C and 25.61 °C, respectively, with total precipitations of approximately 725.29 mm and 1058.4 mm.

**Table 1:** Basic soil properties at the experimental site.

soil category	Alkali-hydrolyzable nitrogen (mg·kg^-1^)	Available Phosphorus (mg·kg^-1^)	Extractable Potassium (mg·kg^-1^)	Total Nitrogen (g·kg^-1^)	Total Phosphorus (g·kg^-1^)	Total Potassium (g·kg^-1^)	Organic matter (g·kg^-1^)	pH
Sandy loam	103.9	45.59	113.11	0.37	0.83	10.16	12.75	7.95

### 2.2. Experimental design

**Fig 2:**
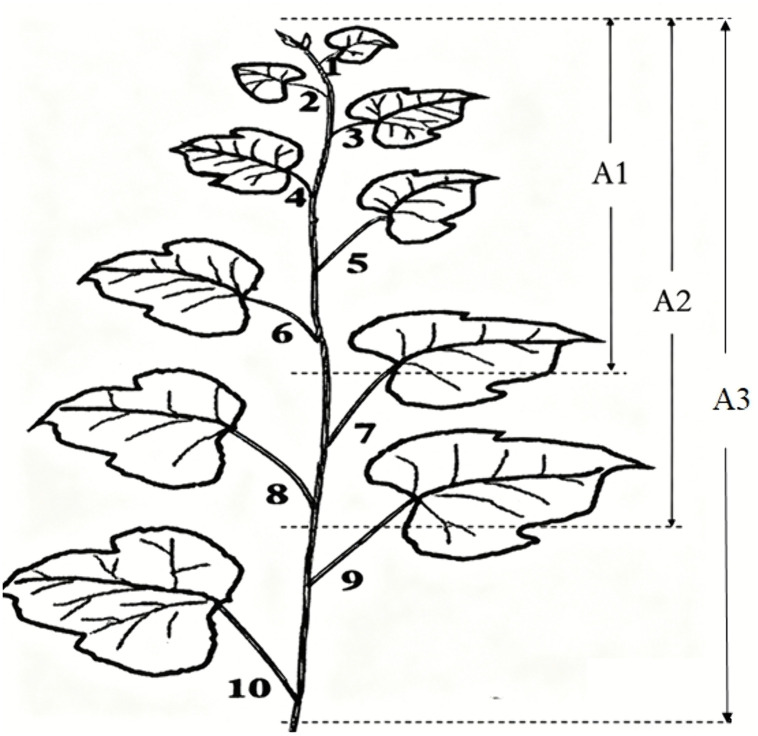
Illustration of sweet potato vine nodes. (A) This is an illustration of sweet potato vine nodes.

**Fig 3:**
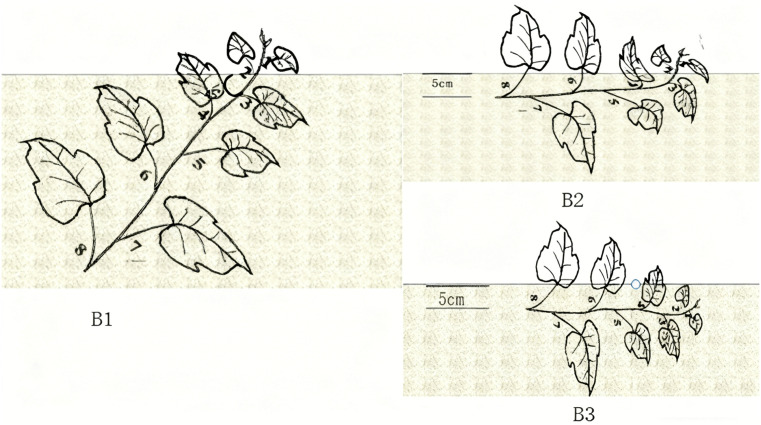
Illustration of sweet potato vine placement in soil. (A) This is an illustration of sweet potato vine placement in soil.

The experiment employed a two-factor split-plot design. The main plots were the node number of vine cuttings (A), with three levels: 6-node seedlings (A1), 8-node seedlings (A2), and 10-node seedlings (A3).The subplots were planting method (B), with three levels: slant planting with exposed tip (B1), flat planting with exposed tip (B2), and flat planting without exposed tip (B3). Among these, A1B1 represents the conventional planting type in production (CK). A total of 9 treatments were set up, each with three replications (Figs 2 and 3).

The trial employed staggered ridge planting, with 4 ridges per plot. Each subplot covered 19.2 m^2^, totaling 100 plants. Ridge width was 80 cm, and plant spacing was 24 cm. Planting occurred on May 15, 2022, and May 15, 2023, with harvest on October 12. Basal application of compound fertilizer (N: P₂O₅: K₂O = 15: 5: 25) at 225 kg·ha^-1^ as basal fertilizer. After transplanting, all plots were irrigated immediately by manual hose irrigation, with water applied directly around the root zone of each plant to ensure seedling establishment. During the growing season, supplementary irrigation was applied when necessary according to rainfall and soil moisture conditions. The same irrigation management was applied uniformly to all treatments, and irrigation was not included as an experimental factor. Other field management practices were conducted according to local conventional cultivation practices. Other field management practices were conventional (Table 2).

**Table 2:** Experimental design.

Factor	Level	Treatment Description	Details
A (Node No.)	A1	6-node seedling	Vine with 6 nodes (Fig 2)
	A2	8-node seedling	Vine with 8 nodes (Fig 2)
	A3	10-node seedling	Vine with 10 nodes (Fig. 2)
B (Planting)	B1	slant planting with exposed tip	Planted at ~45° angle, top 1–2 nodes exposed (Fig 3)
	B2	flat planting with exposed tip	Buried ~5 cm deep, top 1–2 nodes exposed (Fig 3)
	B3	flat planting without exposed tip	Fully buried ~5 cm deep, tip not exposed (Fig 3)

### 2.3. Measurement indicators and methods

#### 2.3.1. Root morphology.

At 20, 30, and 40 days after transplanting (DAT) (during branching and storage root formation), three plants were randomly selected from each plot. Adventitious roots were cut at nodes, pooled, and cleaned of soil using trays. Root images were scanned using a Topo Root Scanner (MICROTEK ScanMaker i800plus, Shanghai, China) to scan the roots. The scanned root images were analyzed using WinRHIZO Pro (Regent Instruments Inc., Quebec, Canada) root analysis software to determine root diameter, root length, and root tips number at each node position for each treatment.

#### 2.3.2. Branch number.

At harvest, at least 30 plants consecutively were selected from each treatment. The number of branches within 30 cm above the base of the main vine (referred to as main stem branches) and the number of branches emerging from nodes on underground vine (referred to as underground node branches) were counted.

#### 2.3.3. Endogenous hormone content in buried nodes.

At 70 DAT (early tuber enlargement stage), three plants were sampled from each plot. Subterranean vines were segmented by node, and samples were collected, stored in liquid nitrogen, and sent to Chongqing Bonoheng Biotechnology Co., Ltd. for measurement of abscisic acid (ABA) and auxin (IAA) content in subterranean internodes using the enzyme-linked immunosorbent assay (ELISA) method. The endogenous hormone contents were determined using commercial ELISA kits (Shanghai Hengyuan Bio-Technology). Briefly, frozen samples were homogenized and extracted using the extraction buffer provided with the kits. After centrifugation, the supernatant was used for ELISA analysis. Absorbance was measured at 450 nm using a microplate reader, and hormone concentrations were calculated according to the standard curves, strictly following the manufacturer’s instructions.

#### 2.3.4. Tuber yield and storage root number.

At 150 DAT, storage roots were harvested by plot for yield measurement. The swollen storage roots were manually separated from fibrous roots and fine adventitious roots. Only swollen storage roots were counted and weighed for the determination of storage-root number and yield. In one replicate, 30 plants per plot were selected to record storage root number per node (RNN) and storage root weight per node (RNW) and calculated the yield and storage root number (RN). The proportion of storage root weight per node (RNWP) and the proportion of storage root number per node (RNNP) were calculated as:


$$\begin{array}{c} RNWP(\%)=(storage\;root\;weight\;at\;node)÷(total\;storage\;root\;weight)\times 100\% \end{array}$$

$$\begin{array}{c} RNNP\left(\%\right)=(number\;of\;storage\;roots\;at\;node)÷(total\;number\;of\;storage\;roots)\times 100\% \end{array}$$

### 2.4. Data analysis

Data statistics and graphing were performed using Excel 2021. A one-way analysis of variance (ANOVA) in SPSS 25.0 software (IBM Corporation, Armonk, NY, USA) was conducted to assess the effect of different treatments. A two-way ANOVA was used to evaluate the effects of the node number of vine cuttings (A), planting method (B), and their interaction on the studied parameters. Correlation analysis was conducted using SPSS 22.0 software (IBM Corp., Chicago, IL, USA). Graphing was performed using GraphPad Prism (GraphPad Software, Boston, Massachusetts, USA). Pearson correlation analysis was performed to determine the relationships among different variables, and the correlation heatmap was generated using Origin 2025.

## 3. Results and analysis

### 3.1. Effects on IAA and ABA content in buried nodes

#### 3.1.1. Overall node IAA and ABA content.

As shown in Table 3, both the number of the node number of vine cuttings and planting method exerted extremely significant effects on IAA and ABA content across all nodes. The interaction between these two factors did not reach the level of significance for IAA and ABA content across all nodes. Compared to A1, A2 and A3 showed significant increases in total IAA content across all nodes by 83.33% and 114.29%, respectively, and in ABA content by 51.13% and 87.73%, respectively. Compared with B1, B2 and B3 showed significant increases in IAA content across all nodes by 5.81% and 30.23%, respectively, while ABA content decreased by 3.23% and increased significantly by 40.32%, respectively.

**Table 3:** The hormone content in buried nodes across treatments in 2023.

Treatment		IAA Content (pmol·g^-1^ FW)	ABA Content (ng·g^-1^ FW)
A1	B1	0.12 ± 0.05 cd	227.36 ± 92.98d
	B2	0.09 ± 0.04d	229.19 ± 94.92d
	B3	0.21 ± 0.06bc	348.74 ± 96.45 cd
A2	B1	0.23 ± 0.07bc	346.94 ± 96.38 cd
	B2	0.23 ± 0.07bc	395.72 ± 110.53bc
	B3	0.31 ± 0.06ab	474.34 ± 83.61abc
A3	B1	0.27 ± 0.05ab	446.32 ± 80.07abc
	B2	0.28 ± 0.06ab	510.09 ± 97.04ab
	B3	0.35 ± 0.05a	555.36 ± 43.56a
Significance	A	**	**
	B	**	**
	A × B	ns	ns

(A) A: Node number of vine cuttings; B: Planting method. Different letters within the same column indicate significant differences at the 0.05 level. * and ** denote significant differences at the 0.05 and 0.01 levels, respectively. ns indicates no significant difference.

#### 3.1.2. Node-specific IAA and ABA content.

Tables 4 and 5 show the percentage of IAA and ABA content at each node relative to the node immediately below ground. Except for the A1B1 and A1B2 treatments at the 4th node below ground, the IAA content at all other nodes increased initially and then decreased as the node number increased. Compared to the IAA content at the node immediately below ground, the IAA content reached its peak at the 4th below ground for all treatments (Table 4). ABA content across nodes showed an initial increase followed by a decrease. Compared to the antepenultimate node, ABA content peaked at the 2th node buried in soil across all treatments. It is speculated that the high IAA content after the 4th node buried in soil may weaken tuber formation capacity at that node, with tuber formation primarily occurring in the first three nodes.

**Table 4:** Proportion of IAA content per node relative to terminal node in 2023 (%).

Node	A1B1	A1B2	A1B3	A2B1	A2B2	A2B3	A3B1	A3B2	A3B3
1	/	/	66.67	/	/	84.00	/	/	105.26
2	/	/	72.22	/	/	148.00	/	/	131.58
3	71.43	65.52	91.67	46.51	54.55	176.00	100.00	95.24	184.21
4	82.86	72.41	125.00	58.14	68.18	224.00	140.00	128.57	305.26
5	97.14	75.86	116.67	86.05	70.45	220.00	195.00	171.43	284.21
6	100.00	100.00	100.00	120.93	120.45	180.00	245.00	276.19	236.84
7	/	/	/	111.63	106.82	112.00	220.00	242.86	215.79
8	/	/	/	100.00	100.00	100.00	210.00	204.76	168.42
9	/	/	/	/	/	/	115.00	114.29	105.26
10	/	/	/	/	/	/	100.00	100.00	100.00
Antepenultimate node IAA (pmol·g^-1^)	0.35	0.29	0.36	0.43	0.44	0.25	0.2	0.21	0.19

(A) A: Node number of vine cuttings; B: Planting method. Numbers 1–10 indicate the first few nodes of sweet potato stems from tip to end. The antepenultimate node refers to the terminal node of the inserted vine.

**Table 5:** Proportion of ABA content at each node from highest to lowest node in 2023 (%).

Node	A1B1	A1B2	A1B3	A2B1	A2B2	A2B3	A3B1	A3B2	A3B3
1	/	/	133.25	/	/	153.60	/	/	227.65
2	/	/	132.70	/	/	170.93	/	/	244.88
3	105.92	120.58	119.99	126.91	149.59	138.98	192.90	241.49	211.99
4	112.96	143.26	107.61	146.84	155.42	130.86	205.34	242.97	210.42
5	103.00	112.54	104.18	123.42	143.75	128.16	181.93	234.86	179.88
6	100.00	100.00	100.00	112.46	130.82	122.20	179.74	219.38	172.83
7	/	/	/	105.98	116.80	118.41	173.88	199.49	160.30
8	/	/	/	100.00	100.00	100.00	165.11	164.12	160.30
9	/	/	/	/	/	/	152.67	153.80	132.11
10	/	/	/	/	/	/	100.00	100.00	100.00
Antepenultimate node ABA (pmol·g^-1^)	538.91	481.09	499.82	484.82	496.9	446.17	330.22	327.8	308.47

(A) A: Node number of vine cuttings; B: Planting method. Numbers 1–10 indicate the first few nodes of sweet potato stems from tip to end. The antepenultimate node refers to the terminal node of the inserted vine.

### 2.2. Effects on root growth under different treatments

#### 2.2.1. Effects on root diameter.

**Fig 4:**
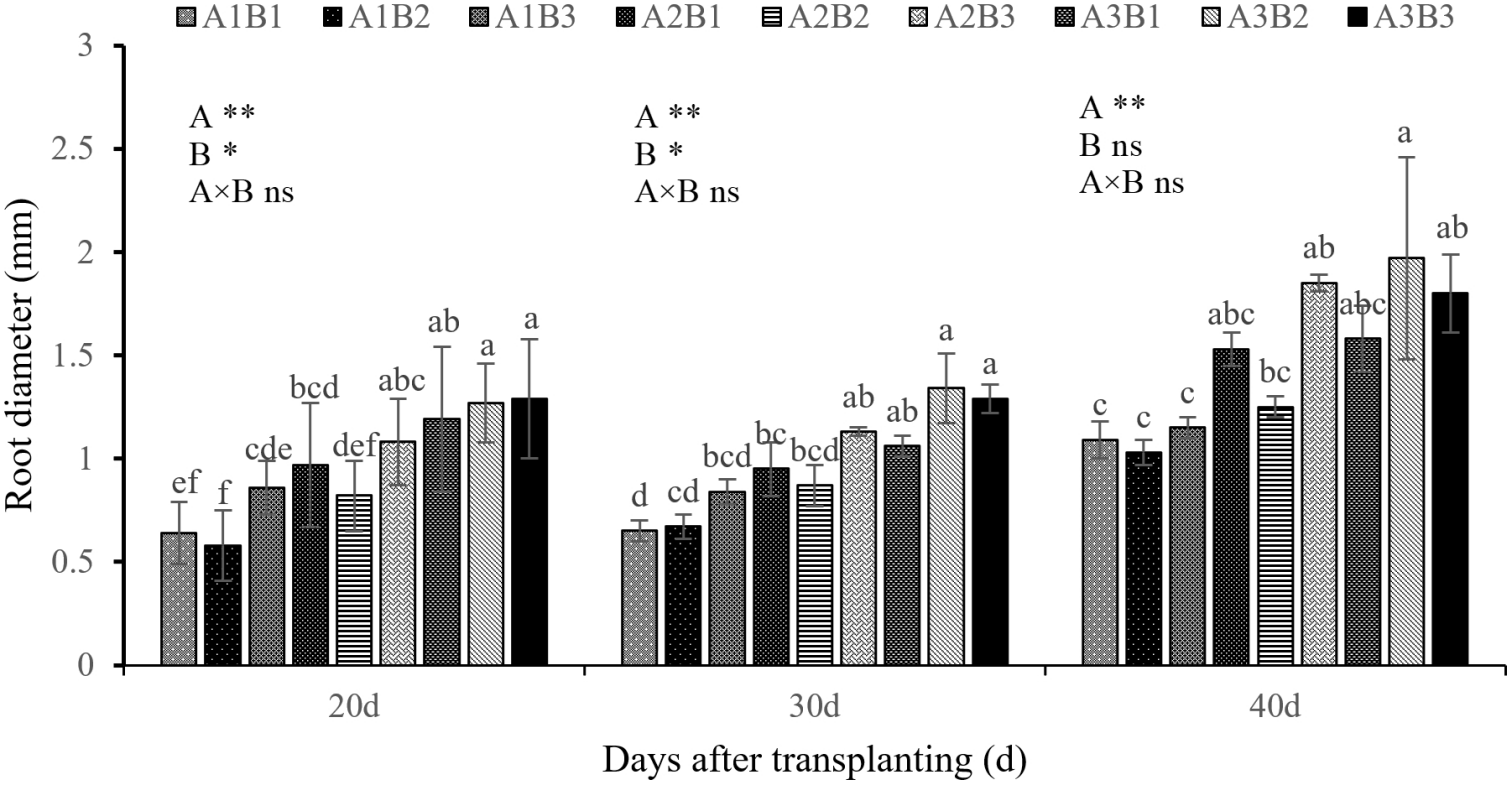
Root diameter at 20, 30, and 40 days after transplanting (DAT) under different treatments in 2023. (A) A: Node number of vine cuttings; B: Planting method. Error bars indicate standard deviation of three replicates. Different letters within the same period indicate significant differences between treatments at the P < 0.05 level. * and ** denote significant differences at the 0.05 and 0.01 levels, respectively. ns indicates no significant difference.

As shown in Fig 4, root diameters at 20, 30, and 40 DAT exhibited the pattern A3 > A2 > A1 across stem node treatments. Compared to treatment A1, treatments A2 and A3 showed significantly increased root diameters at 20, 30, and 40DAT by 37.14%,78.57%, and 36.11%; 70.83%, 41.28%, and 63.30%, respectively. Significant differences existed among the three treatments at 20 and 30 DAT. Regarding planting methods, performance varied across the three time points: at 20d, B3 was significantly higher than B1 and B2; at 30d, B3 was significantly higher than B1.

#### 2.2.2. Effects on root length.

**Fig 5:**
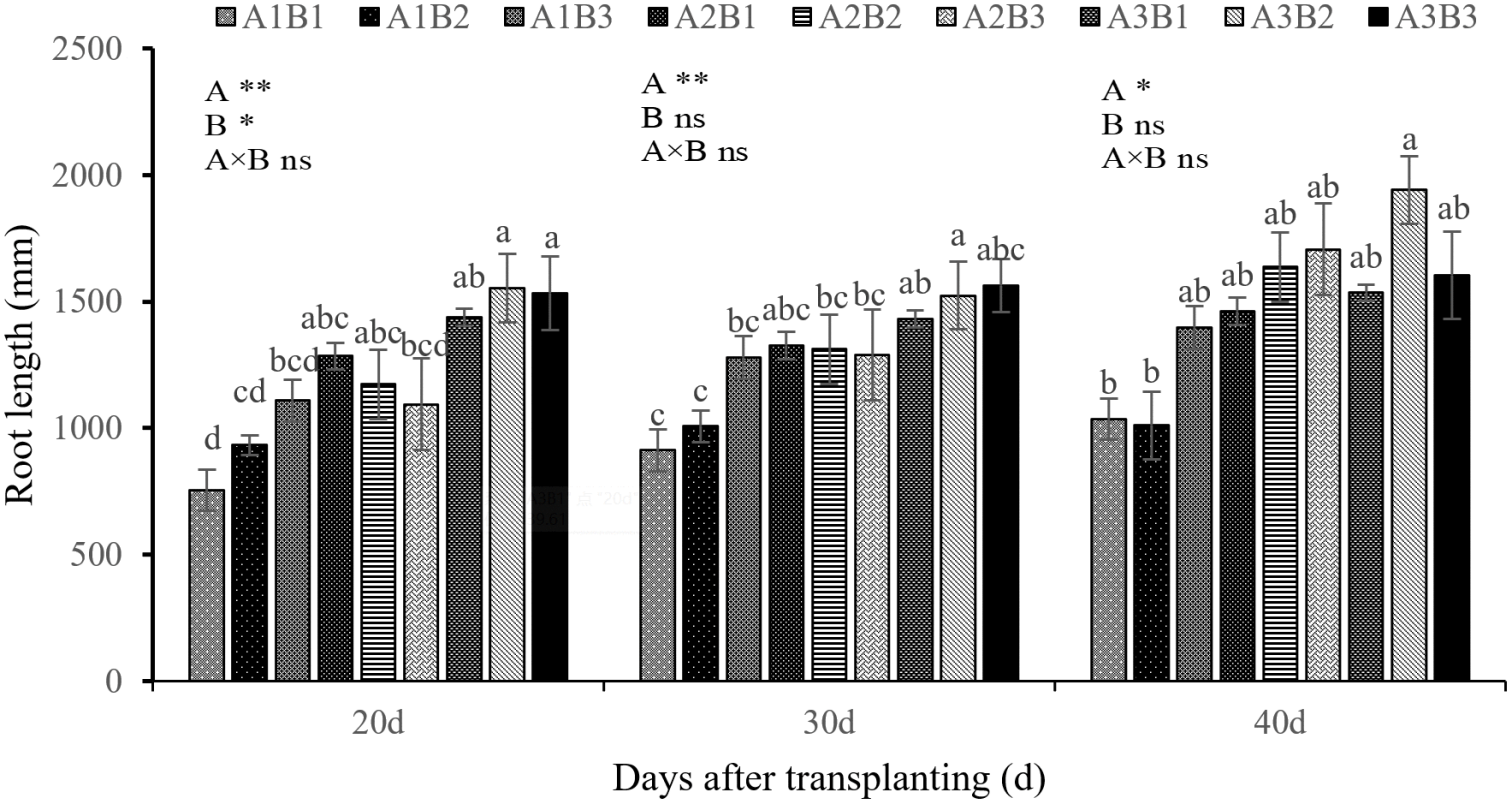
Root length at 20, 30, and 40 days after transplanting under different treatments in 2023. (A) A: Node number of vine cuttings; B: Planting method. Error bars indicate standard deviation of three replicates. Different letters within the same period indicate significant differences between treatments at the P < 0.05 level. * and ** denote significant differences at the 0.05 and 0.01 levels, respectively. ns indicates no significant difference.

As shown in Fig 5, root length at 20, 30, and 40 DAT exhibited the pattern A3 > A2 > A1 across root node count. Compared to treatment A1, treatments A2 and A3 demonstrated significantly increased root lengths at 20d, 30d, and 40d by 27.09%,61.92%, and 22.86%; 41.27%, 39.48%, and 47.42%, respectively. Among planting methods, root length at 20 DAT showed B3 > B2 > B1, with significant differences between B3 and B1.

#### 2.2.3. Effects on root tips number.

**Fig 6:**
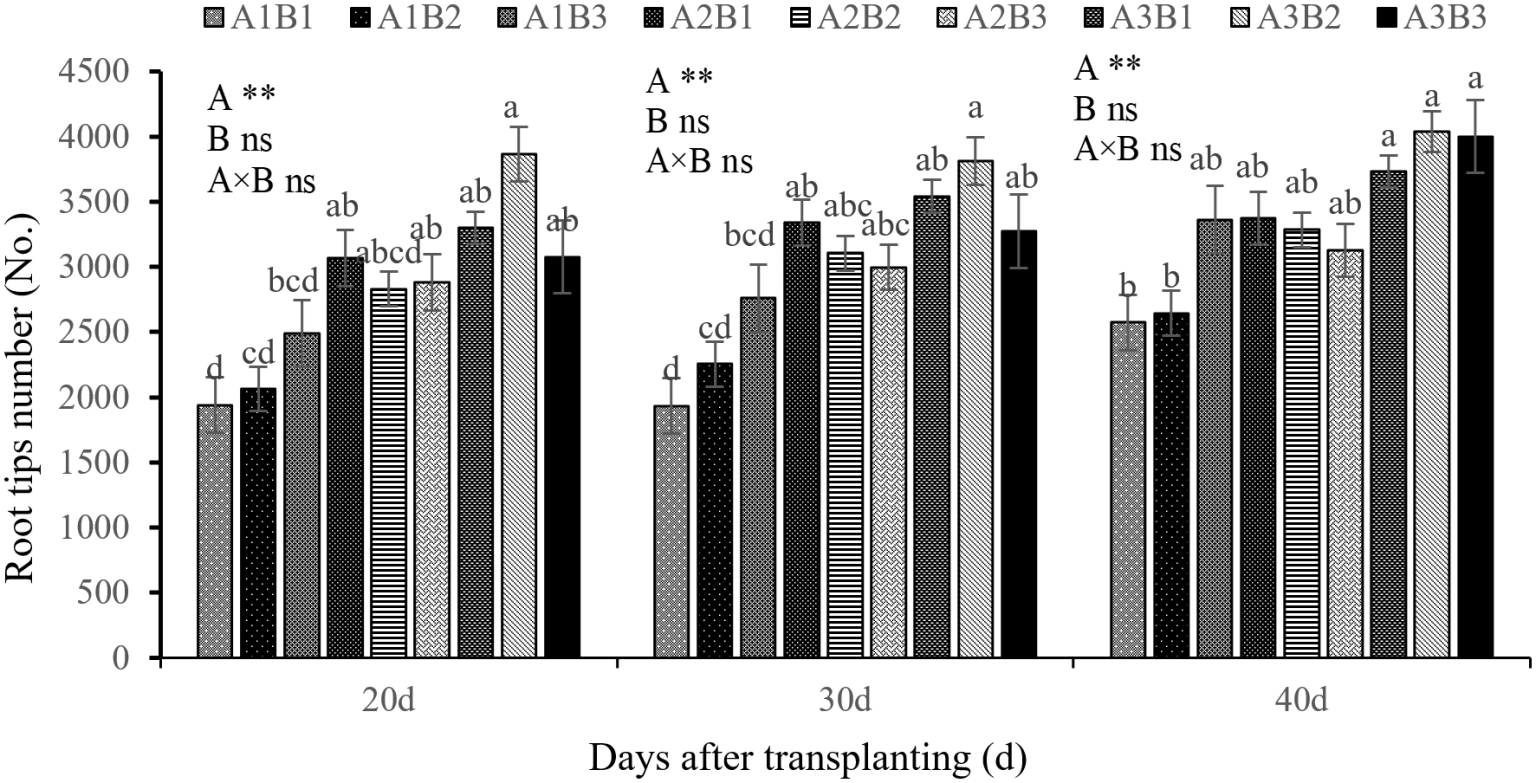
Root tips number at 20, 30, and 40 days after transplanting under different treatments in 2023. (A) A: Node number of vine cuttings; B: Planting method. Error bars indicate standard deviation of three replicates. Different letters within the same period indicate significant differences between treatments at the P < 0.05 level. * and ** denote significant differences at the 0.05 and 0.01 levels, respectively. ns indicates no significant difference.

As shown in Fig 6, root tip numbers at stem node intervals across the three periods exhibited the pattern A3 > A2 > A1, with significant differences among them. Compared to the A1 treatment, the A2 and A3 treatments showed significantly increased root tip numbers at 20d and 40d, specifically by 36.96%, 57.64%, 35.93%, 52.95%, 14.02%, and 37.12%, respectively.

### 2.3. Effects on branching number under different treatments

**Fig 7:**
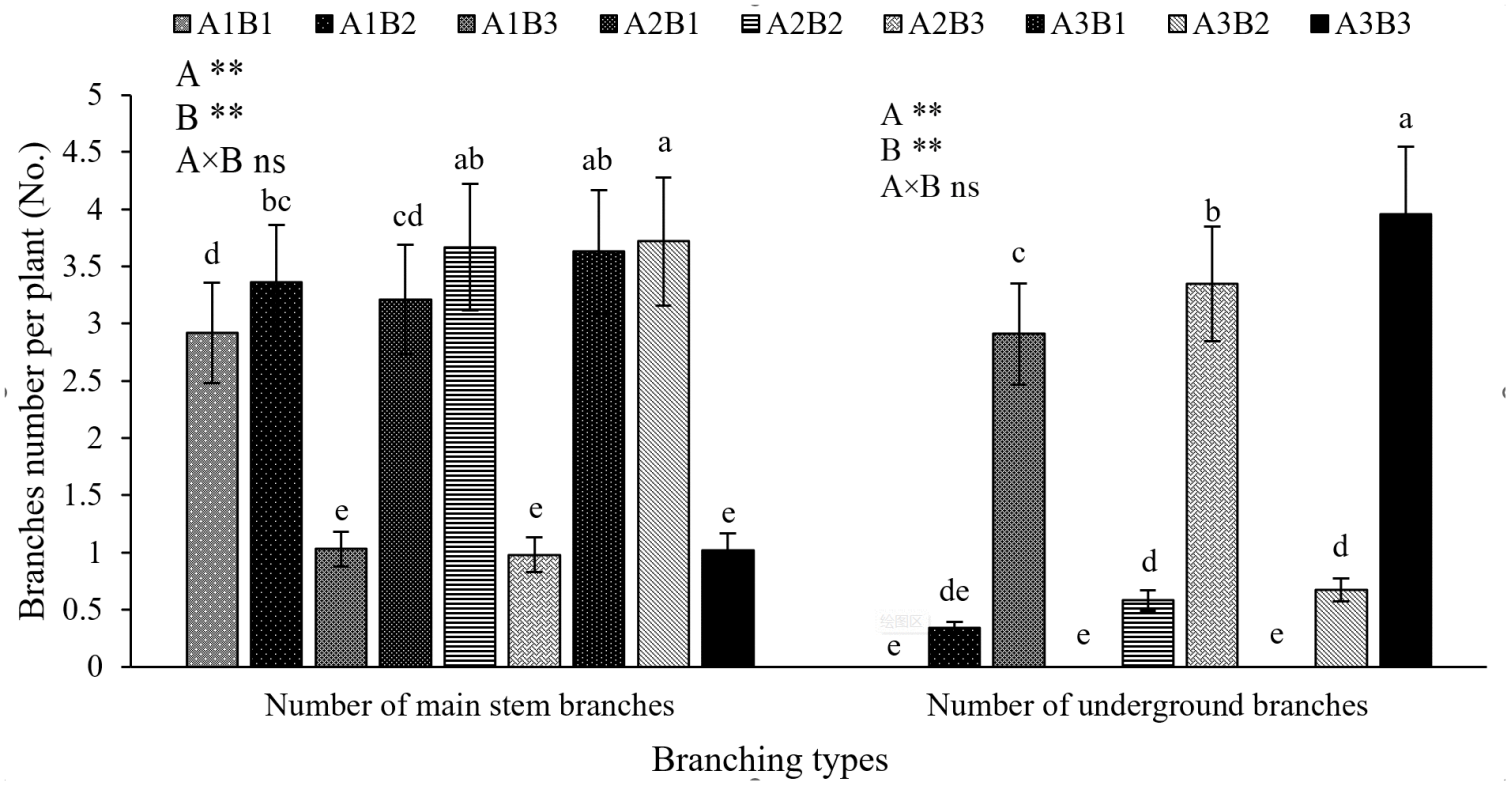
Number of branches per treatment in 2023. (A) A: Node number of vine cuttings; B: Planting method. Error bars indicate standard deviation of three replicates. Different letters within the same period indicate significant differences between treatments at the P < 0.05 level. * and ** denote significant differences at the 0.05 and 0.01 levels, respectively. ns indicates no significant difference.

As shown in Fig 7, both the node number of vine cuttings and planting method exerted extremely significant effects on the number of main stem branches and underground branches. However, their interaction did not significantly influence either the number of main stem branches or underground branches. Compared with A1, the main stem branching number in treatments A2 and A3 increased significantly by 7.52% and 14.50%, respectively. The underground branching number increased significantly by 20.92% and 42.46%, respectively. Compared with B1, the main stem branching number in treatments B2 and B3 increased significantly by 10.15% and decreased significantly by 68.92%, respectively. No underground branching formed in treatment B1.

### 2.4. Effects on tuberization under different treatments

#### 2.4.1. Differences in yield and tuber number among treatments.

As shown in Table 6, both the number of stem nodes and planting method significantly and extremely significantly influenced total sweet potato yield and RN over the two years. However, their interaction only showed an extremely significant effect on total yield in both years. Compared to treatment A1, treatments A2 and A3 significantly increased total yield by 18.58% and 29.04% in 2021, and by 28.79% and 31.11% in 2022, respectively. RN in 2021 increased significantly by 19.01% and 26.54%, respectively, and in 2022 by 42.40% and 52.09%, respectively. This indicates that the number of nodes buried underground significantly influenced both yield and RN, with both increasing as the number of underground nodes increased.

**Table 6:** Multiple comparisons of total sweet potato yield and total tuber number per treatment in 2022 and 2023.

Treatment		2022		2023	
		Yield (kg·667m^-2^)	RN (No.·667m^-2^)	Yield (kg·667m^-2^)	RN (No.·667m^-2^)
A1	B1	2333.61 ± 299.53f	19717.35 ± 1200.66e	1914.31 ± 105.33f	15711.56 ± 380.16f
	B2	2535 ± 243.28ef	22378.08 ± 1504.55de	2190.54 ± 99.66e	20215.86 ± 1547ef
	B3	2699.75 ± 296.32de	23636.08 ± 1963.93cde	2752.26 ± 206.71 cd	24773.7 ± 1426.28cde
A2	B1	2528.49 ± 248.4ef	21213.52 ± 1787.7e	2611.23 ± 97.98d	24001.71 ± 2372.42de
	B2	3124.31 ± 256.62bc	27898.25 ± 2863.9abc	3089.85 ± 43.59ab	30990.79 ± 1406.74ab
	B3	3321.84 ± 348.01ab	29115.45 ± 2614.37ab	3130.26 ± 57.64ab	31445.76 ± 1643ab
A3	B1	2873.52 ± 213.26cde	24083.48 ± 1922.25bcd	2814.74 ± 70.89 cd	27421.11 ± 1780.21bcd
	B2	3907.22 ± 465.89a	31902.52 ± 4747.6a	3260.7 ± 165.66a	35031.91 ± 2433.61a
	B3	2985.83 ± 264.42bcd	27190.3 ± 2317.95abcd	2915.16 ± 25.89bc	29866.78 ± 546.28bc
Significance	A	**	*	**	**
	B	**	*	**	**
	A × B	**	ns	**	ns

(A) A: Node number of vine cuttings; B: Planting method. Different letters within the same column indicate significant differences at the 0.05 probability level. RN: Storage root number. * and ** indicate significant differences at the 0.05 and 0.01 levels, respectively. ns indicates no significant difference.

#### 2.4.2. Effects of different treatment combinations on tuberization at various node positions.

As shown in Tables 7 and 8, the yield at the inverted node exhibited the pattern A1 > A2 > A3, while the number of storage root at the inverted node followed B2 > B1 > B3. Although increasing buried nodes by altering the node number of vine cuttings or planting methods expanded the range of nodes producing tubers underground, yield and storage root setting contributions remained concentrated in a few key nodes. For treatments B1 and B2, the first buried node contributed the highest yield proportion, which decreased with increasing burial depth. Treatment B3 showed the highest yield contribution from the second buried node, exhibiting an overall trend of initial increase followed by decline. The number of storage roots formed per treatment showed an initial increase followed by a decrease with increasing burial depth, with the 2th node consistently contributing the highest proportion. Treatments B2 and B3 promoted more balanced tuber formation across nodes. While increasing the number of buried nodes reduced the contribution of certain nodes, it enhanced overall storage root weight and total storage root count.

**Table 7:** Yield proportion at different nodes under various treatment combinations in 2023(%).

Node	A1B1	A1B2	A1B3	A2B1	A2B2	A2B3	A3B1	A3B2	A3B3
1	/	/	26	/	/	20	/	/	20
2	/	/	31	/	/	30	/	/	25
3	40	39	19	42	28	19	30	28	15
4	30	28	12	21	25	14	26	27	13
5	19	21	8	12	24	8	19	14	10
6	12	12	4	9	12	3	13	11	6
7	/	/	/	9	7	3	4	6	4
8	/	/	/	7	5	3	4	6	2
9	/	/	/	/	/	/	2	4	2
10	/	/	/	/	/	/	1	4	2
Antepenultimate node storage root weight (kg·667m^-2^)	225.97	256.28	123.67	175.06	153.11	79.76	27.31	115.98	45.92

(A) Numbers 1–10 represent the sequential nodes from the apex to the base of the sweet potato vine. The antepenultimate node refers to the terminal node of the inserted vine. RNWP (%) = (storage root weight at node) ÷ (total storage root weight)× 100%.

**Table 8:** Storage root number proportion at different nodes under various treatment combinations in 2023 (%).

Node	A1B1	A1B2	A1B3	A2B1	A2B2	A2B3	A3B1	A3B2	A3B3
1	/	/	19	/	/	7	/	/	17
2	/	/	24	/	/	14	/	/	19
3	29	25	18	19	23	27	24	21	14
4	31	34	13	35	24	16	26	21	13
5	21	21	13	16	21	14	19	17	12
6	19	20	12	13	15	10	11	12	7
7	/	/	/	8	10	7	5	7	6
8	/	/	/	9	7	5	5	8	4
9	/	/	/	/	/	/	5	7	4
10	/	/	/	/	/	/	4	6	3
Antepenultimate node storage root number (No.·667m^-2^)	2946	4043	3028	2250	2284	1469	1143	2229	996

(A) Numbers 1–10 indicate the node positions from the tip to the base of the sweet potato stem. The antepenultimate node refers to the terminal node of the inserted vine. RNNP (%) = (number of storage roots at node)÷(total number of storage roots)× 100%

### 2.5. Correlation analysis

**Fig 8:**
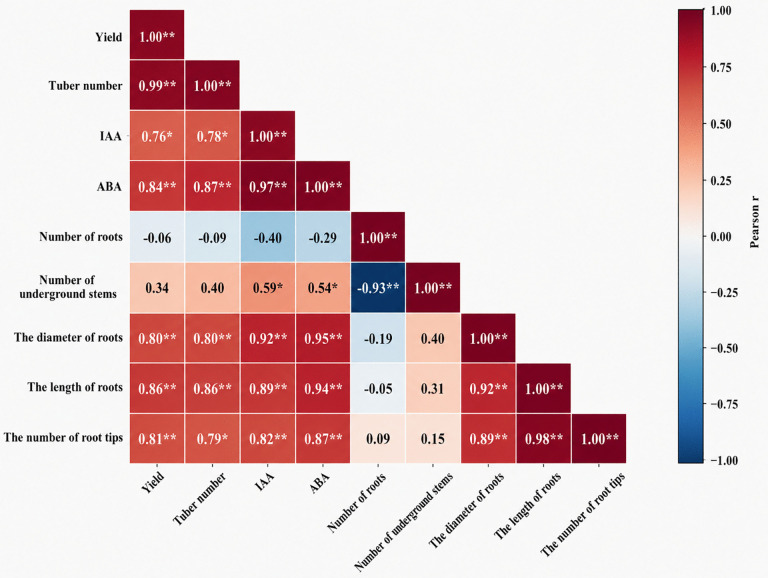
Correlation analysis. A. Correlation analysis among yield, storage root number, hormone contents, vine branching traits, and root morphological traits of sweet potato. (B)The values in the squares represent Pearson correlation coefficients. The color scale indicates the direction and magnitude of correlations. * and ** indicate significant correlations at P < 0.05 and P < 0.01, respectively.Reference. PDF version of the references.

Pearson correlation analysis was conducted to elucidate the relationships among yield, morphological traits, and endogenous hormones (Fig 8). The results revealed that total yield exhibited highly significant positive correlations (*P* < 0.01) with storage root number (*r* = 0.99), root length (*r* = 0.86), root diameter (*r* = 0.80), and root tips number (*r* = 0.81). Furthermore, the endogenous hormones IAA and ABA were strongly positively correlated with both yield (*r* = 0.76 and *r* = 0.84, respectively) and underground root morphological traits. Conversely, main vine branching showed no significant correlation with yield (*r* = −0.06) but demonstrated a strong negative correlation with underground vine branching (*r* = −0.93).

## 3. Discussion

### 3.1. Effects of the node number of vine cuttings on storage root formation capacity in sweet potato

The number of nodes on sweet potato vine cuttings is a key factor affecting yield. This study showed that compared to 6-node cuttings, 8-node and 10-node cuttings significantly increased total storage root weight and total storage root count, consistent with findings by Hall et al.[21] and Mo [22]. This advantage may be related not only to the increased number of buried nodes, which provide more potential sites for adventitious root emergence, but also to stronger early root establishment. Within 30–40 DAT, 8-node to 10-node cuttings exhibited significantly superior root length, root tip count, and root diameter compared to 6-node cuttings. This finding aligns with the research by Wan Haiqing et al. [23]. Early root differentiation and development provide more ample storage space for storage root formation, thereby enhancing tuber yield [9,10,24]. Regarding aboveground growth, the number of branches in 8-node and 10-node seedlings significantly increased, which may improve photosynthetic efficiency before canopy closure and facilitating greater dry matter translocation to tubers [9,25]. However, the yield advantage of longer cuttings should not be attributed to a single factor. Instead, it likely results from the coordinated effects of buried-node number, early adventitious root development, aboveground growth, and assimilate allocation.

As stem node numbers increased, endogenous hormone levels of ABA and IAA were upregulated. This suggests that longer vine cuttings may alter the hormonal status of buried nodes during early storage root enlargement. IAA is closely associated with cell division, cambial activity, and vascular development, whereas ABA may be involved in assimilate accumulation, stress response, and sink activity [19,20,26]. Therefore, the higher ABA and IAA levels observed in 8- and 10-node cuttings may be associated with enhanced storage root enlargement and sink strength. Overall, 8–10 node seedlings increase yield primarily by promoting early root establishment, enhancing aboveground assimilation capacity, and optimizing the hormonal environment to increase tuber set.

### 3.2. Effects of planting method on sweet potato storage root formation capacity

Planting method is a critical factor in regulating sweet potato yield. This study found that flat planting significantly increased both total yield and total tuber count compared to slanting planting with exposed tips, consistent with most research conclusions [28–29]. The yield-enhancing mechanism of flat planting is primarily manifested in the regulation of aboveground growth [30]. Flat planting with exposed tips rapidly establishes above-ground biomass, enhancing photosynthetic capacity earlier [31]. Conversely, flat planting without exposed tips delays the emergence of the terminal bud but significantly promotes the growth of underground node branches [32], establishing more efficient pathways for assimilate transport. This facilitates biomass compensation and optimized allocation during the canopy closure stage [30–33].

At 70 DAT, flat planting significantly increases ABA content at the same buried node and overall IAA levels. ABA promotes tuber differentiation by activating amylose synthase [20], while IAA synergistically regulates cell enlargement [15–16], collectively expanding the “sink capacity.” Simultaneously, flat planting increases yield by boosting tuber number per plant, aligning with the principle that “tuber number drives yield” [27–29]. The impact of planting method on root systems is evident only in the early stages, with no significant differences after 40 days, indicating that yield enhancement hinges on optimizing “source-sink-flow” coordination rather than root morphology [19,29,34].

### 3.3. Effects of different treatments on nodal storage root formation capacity in sweet potato

This study indicated that the capacity of storage root formation varies significantly among different internodes buried in soil, jointly regulated by the nodes number of storage root and planting method. The second buried internode exhibited stronger tuber formation capacity, while basal internodes showed progressively reduced capacity. This correlates closely with differences in the timing of adventitious root development, initial growth rate, and differentiation toward tuber formation among these internodes [10,24].

At 70 DAT, ABA and IAA contents varied among buried nodes. The relatively high ABA content at the second buried node and the node-dependent distribution of IAA suggest that the hormonal status of subterranean internodes may be associated with nodal differences in storage root enlargement. IAA is closely related to cell division, cambial activity, and vascular development, whereas ABA may be involved in assimilate accumulation and sink activity during storage root enlargement [10,20].Increasing the node number of vine cuttings promotes root length, root diameter, and root tip number, providing a robust foundation for nutrient absorption essential for multi-node tuber formation [23]. Although the 7th and 8th nodes themselves contribute a low proportion of tubers, they influence overall tuber formation by regulating the systemic signaling pathways governing the tuberization process [35–36]. Therefore, in production, using 8–10-node seedlings combined with flat planting (A2/A3) can synergistically optimize hormone distribution, root development, and assimilate allocation, enhancing the contribution of multiple buried nodes to storage root formation.

In addition, only one cultivar, Yuhongxinshu 98, was used in this study, and ABA and IAA contents were measured only at 70 DAT, corresponding to the early storage-root enlargement stage. Therefore, the observed hormone response patterns may be cultivar-dependent and mainly reflect physiological responses during early storage-root enlargement rather than storage-root initiation. Future studies involving multiple cultivars and earlier multi-stage hormone measurements are needed to clarify the generality of these hormonal responses and the dynamics of hormone regulation during storage-root initiation and enlargement.

## 4. Conclusion

Increasing stem node numbers can elevate endogenous hormone levels within buried nodes, upregulate overall ABA and IAA levels in the underground system, promote early root establishment, increase underground branching, and ultimately boosts yield and storage root count. Flat planting can overcome the apical dominance mediated by ABA, enhance storage root formation potential in middle-to-lower buried nodes, and stimulate root growth and underground branching to increase yield and storage root count. In summary, the 8-node seedling combined with flat planting (A2/A3) represents the optimal planting pattern. This approach can synergistically optimize hormone distribution, fully unlock tuber-setting potential at all nodes, and achieve significant increases in both yield and storage root count, providing theoretical and technical support for high-yield sweet potato cultivation.
